# How to Recondition *Ex Vivo* Initially Rejected Donor Lungs for Clinical Transplantation: Clinical Experience from Lund University Hospital

**DOI:** 10.1155/2011/754383

**Published:** 2011-08-24

**Authors:** Sandra Lindstedt, Atli Eyjolfsson, Bansi Koul, Per Wierup, Leif Pierre, Ronny Gustafsson, Richard Ingemansson

**Affiliations:** ^1^Department of Cardiothoracic Surgery, Lund University and Skåne University Hospital, Lund, Sweden; ^2^Department of Cardiothoracic Surgery, Heart and Lung Centre, Lund University Hospital, SE-221 85 Lund, Sweden

## Abstract

A major problem in clinical lung transplantation is the shortage of donor lungs. Only about 20% of donor lungs are accepted for transplantation. We have recently reported the results of the first six double lung transplantations performed with donor lungs reconditioned *ex vivo* that had been deemed unsuitable for transplantation by the Scandiatransplant, Eurotransplant, and UK Transplant organizations because the arterial oxygen pressure was less than 40 kPa. The three-month survival of patients undergoing transplant with these lungs was 100%. One patient died due to sepsis after 95 days, and one due to rejection after 9 months. Four recipients are still alive and well 24 months after transplantation, with no signs of bronchiolitis obliterans syndrome. The donor lungs were reconditioned *ex vivo* in an extracorporeal membrane oxygenation circuit using STEEN solution mixed with erythrocytes, to dehydrate edematous lung tissue. Functional evaluation was performed with deoxygenated perfusate at different inspired fractions of oxygen. The arterial oxygen pressure was significantly improved in this model. This *ex vivo* evaluation model is thus a valuable addition to the armamentarium in increasing the number of acceptable lungs in a donor population with inferior arterial oxygen pressure values, thereby, increasing the lung donor pool for transplantation. In the following paper we present our clinical experience from the first six patients in the world. We also present the technique we used in detail with flowchart.

## 1. Background

Much effort has been devoted worldwide to increasing lung donor availability. An approach that has met with some success has been the extension of criteria used to select donors, for example, including those older than 55 years, with a smoking history of more than 20 pack years, and an abnormal chest roentgenogram, a positive Gram stain for sputum or bronchoalveolar lavage samples, or prolonged mechanical ventilation. Despite this, people are dying while waiting for lung transplants, and there is thus an urgent need to increase the availability of donor lungs [1–3].

In many brain-dead donors, the arterial oxygen tension deteriorates to unacceptable values before organs can be harvested [4, 5]. Edematous lung tissue, due to excessive intravenous infusion in an attempt to maintain adequate donor circulation, may contribute to the deterioration of the gas exchange capacity. Replacement of blood lost during the donor operation with crystalloid solutions will further hemodilute the donor and reduce the plasma colloid osmotic (oncotic) pressure [4, 5]. Due to their spongy structure, the lungs are especially vulnerable to edema under these conditions [4, 6].

Steen and colleagues have developed a new method for *ex vivo* lung evaluation [7, 8], which was used successfully for the first time in humans, when a lung from a non-heart-beating donor was transplanted at the Department of Cardiothoracic Surgery at Lund University Hospital, Sweden, in 2000 [8]. The method can also be used for reconditioning of marginal and nonacceptable donor lungs [9], thus increasing the number of potential lungs available for transplantation. We have recently reported the results of the first six double lung transplantations performed with donor lungs, reconditioned *ex vivo*, that were rejected for transplantation by the Scandiatransplant, Eurotransplant, and UK Transplant organizations [10]. The six patients underwent double sequential lung transplantation with initially rejected donor lungs after reconditioning *ex vivo*. All six double lungs had an initial arterial oxygen pressure below 40 kPa during ventilation at 100% oxygen, and a positive end-expiratory pressure (PEEP) of 5 cm H_2_O before lung harvesting. The criteria for lungs chosen to undergo reconditioning were the same as for conventional donor lungs, except that a lower partial pressure of oxygen in arterial blood (PaO_2_) was accepted. To be accepted for transplantation after reconditioning, the PaO_2_ at a fraction of inspired oxygen (FiO_2_) of 1.0 was required to be 50 kPa or higher, instead of the normal 40 kPa. Three-month survival was 100%. One patient has since died from sepsis (after 95 days), and one from rejection after nine months. Four patients are alive and well two years after transplantation, without any signs of bronchiolitis obliterans syndrome [10, 11].

In this paper we present our clinical experience from the first six patients in the world to receive such reconditioned lungs. We also present the technique used in detail with flowcharts.

## 2. Setup and Priming

**Figure 1:**
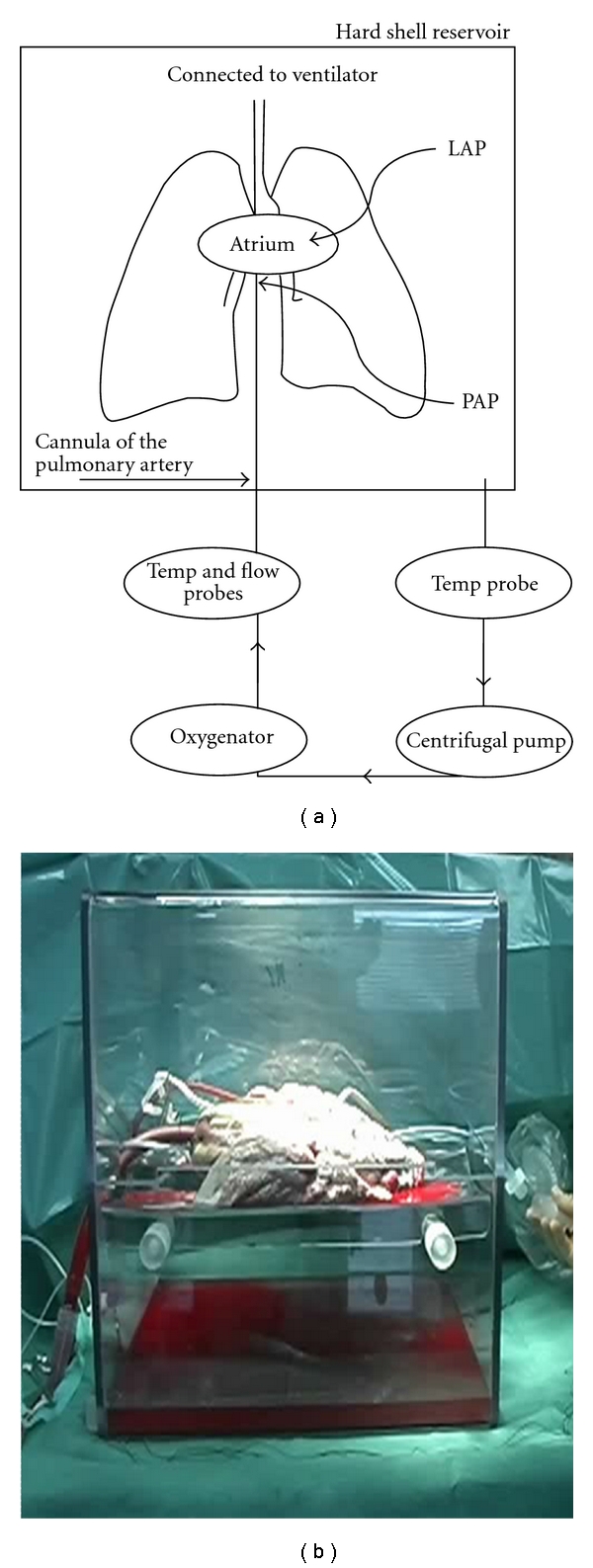
Flowchart showing the first part of the *ex vivo *reconditioning of donor lungs initially rejected donor lungs for clinical transplantation. During the first part of the process, the lungs are not ventilated and the perfusate blood is oxygenated. The temperature of the perfusate is gradually increased with a gradient ≤10°C to 32°C. The initial perfusion flow was 50–100 mL/min. The flow was slowly increased, and the pulmonary pressure was kept at ≤20 mmHg. The left atrium pressure was maintained at 0 mmHg to prevent the development of edema.

Figure 1(a) shows the perfusion circuit, which consists of a hard-shell reservoir (Medtronic Nederland, Kerkrade, the Netherlands), a centrifugal pump (Biomedicus, Bio-Console 550; Electromedics Inc., Grand Rapids, MI), a membrane oxygenator with a built-in heat exchanger (Qadrox HMO 1011; Jostra AG, Germany), and a leukocyte/arterial filter (LG 6; Pall, Newquay, Cornwall, UK). A flow probe (Bio Probe TX50; Electromedics Inc.), Polytrode oxygen sensor (pO_2_; Polystan, Værløse, Denmark), and temperature sensors (Space Labs Medical Inc., Redmond, Wash USA) are also included. A photograph of the setup is shown in Figure 1(b).

**Figure 2:**
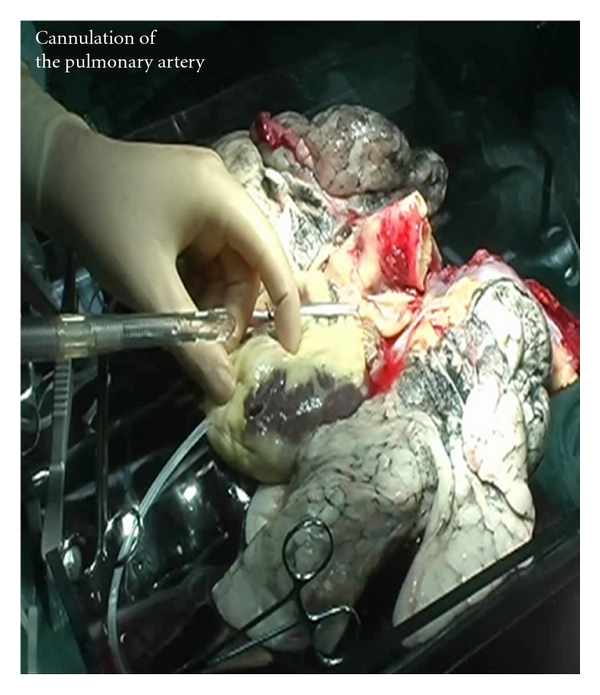
Schematic (a) and photograph (b) of the *ex vivo* lung reconditioning and evaluation system. The blood leaving the remaining dorsal part of the left atrium runs freely out into the box containing the lung. The pulmonary arterial pressure (PAP) is measured continuously. The left atrium pressure (LAP) is maintained at 0 mmHg. Blood gases are measured in the blood before and after the lung. The system is primed with STEEN solution and erythrocyte concentrate.

A 28-French venous cannula was placed with its tip in the main pulmonary artery (Figure 2). A baby feeding catheter was placed in the pulmonary artery to measure the perfusion pressure. During reconditioning, the perfusion solution flowed directly out into the lung reconditioning box. Therefore, the left atrium pressure was zero. Before starting perfusion, the pulmonary artery cannula was connected to the corresponding tube of the extracorporeal circuit, the air was removed, and the shunt of the circuit was clamped.

The system was primed with 2 liters of STEEN solution (Vitrolife AB, Lund, Sweden), mixed with 2 units of ABO-compatible erythrocyte concentrate, that had been irradiated, leukocyte filtered and washed, to a hematocrit of 15%. STEEN solution is a physiological electrolyte solution containing human serum albumin and dextran. This solution is a buffered, extracellular solution with a composition designed to provide a colloid osmotic pressure allowing the physiological pressure and flow to be maintained without the development of pulmonary edema [5, 7, 8, 10, 12].

Imipenem (0.5 g, Tienam, Merck Sharp & Dohme, Sollentuna, Sweden), insulin (20 IU, Actrapid, Novo Nordisk, Bagsvaerd, Denmark), and heparin (10,000 IU, Leo Pharma, Malmö, Sweden) were added, and isotonic trometamol (Addex-Tham, Kabi, Sweden) was used to buffer the mixed solution to a temperature-adjusted pH of 7.4 [7, 8, 10, 12].

## 3. Reconditioning Part 1

**Figure 3:**
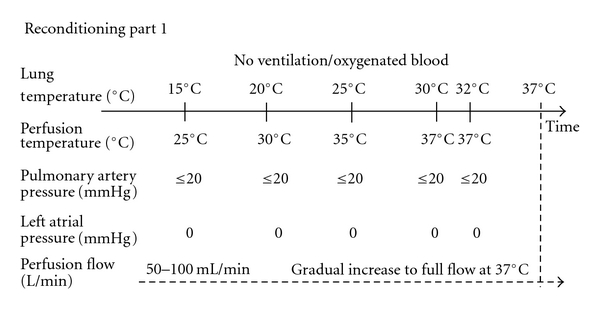
Flowchart showing the second part of the *ex vivo* reconditioning of donor lungs initially rejected donor lungs for clinical transplantation. During the second part of the process, the lungs are ventilated and the perfusate blood is oxygenated. When the temperature reaches 32°C, careful ventilation is started at 1 L/min. The ventilation rate is increased with one liter per increasing degree of perfusate from the lung (i.e., increasing lung temperature). The FiO_2_ was maintained at 1.0, and the respiratory frequency was gradually increased from 5 to 15–20 per min. The perfusion flow was limited by the pulmonary pressure (≤20 mmHg). The PEEP was gradually increased to 5 cm H_2_O at 37°C.

Figure 3 shows a flowchart of the first part of the reconditioning process, during which the lungs are not ventilated, and the perfusate blood is oxygenated. After removing the air from the pulmonary artery, slow perfusion (perfusion pressure ≤ 20 mmHg) was started at 25°C. The initial temperature of the perfusate was 25°C, since the temperature of the lungs, after taking them out of the box with Perfadex solution, and after connecting them to the *ex vivo* circuit, is approximately 15°C, and the gradient between the lung temperature and the temperature of the perfusate is kept at approximately 10°C. The temperature in the perfusate was gradually increased to 32°C with a gradient of ≤10°C. The initial perfusion flow was 50–100 mL/min. The flow was slowly increased, and the pulmonary pressure was kept at ≤20 mmHg. The left atrium pressure was maintained at 0 mmHg to prevent the development of edema.

## 4. Reconditioning Part 2

**Figure 4:**
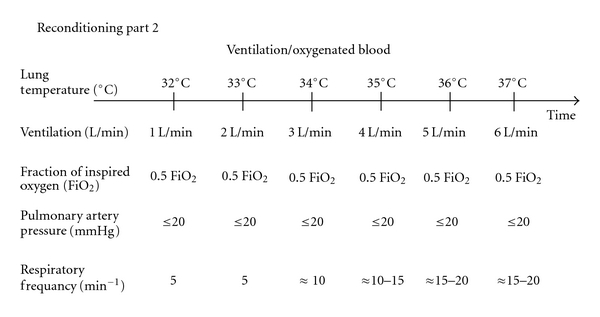
Flowchart illustrating the evaluation of *ex vivo* reconditioned lungs for clinical transplantation. During the evaluation, the lung is fully ventilated and the perfusate blood is deoxygenated. The gases entering the oxygenator are mixed to obtain values of pCO_2_ of 4.5–5 kPa. At this point, the perfusion flow was 4–6 L/min (i.e., full flow). Samples of the perfusate blood are taken before and after passing through the lung (i.e., venous and arterial blood samples) after 5 minutes' exposure to FiO_2_ of 0.21, 0.5, and 1.0 in order to measure blood gases. A deflation test is then performed. If the deflation test is normal and the PaO_2_ with FiO_2_ = 1.0 was 50 kPa or higher, the lungs are accepted for transplantation.

Figure 4 shows a flowchart describing the second part of the reconditioning process, during which the lung is ventilated, and the perfusate blood is oxygenated. Careful ventilation of the lungs was started (1 L/min) when the temperature of the perfusate leaving the lungs had stabilized at 32°C, as the ventilation of cold lungs may lead to lung membrane injury [5]. The rate of ventilation was increased by one liter per minute for every degree increase in temperature of the perfusate leaving the lungs (i.e., the lung temperature) up to 37°C, at which normal ventilation (100 mL/kg/min) was reached. The gases entering the oxygenator were mixed to obtain temperature-corrected perfusate gas values of pCO_2_ of 4.5–5 kPa. The FiO_2_ was maintained at 1.0, and the respiratory frequency was gradually increased from 5 to 15–20 per min. The perfusion flow was gradually increased and reached full flow (5-6 L/min) at 37°C. The perfusion flow was limited by the pulmonary pressure. The pulmonary pressure was maintained at ≤20 mmHg. When the perfusate from the lung reached 37°C, normal ventilation (100 mL·kg^−1^·min^−1^) was given. The PEEP was gradually increased and reached 5 cm H_2_O at 37°C.

## 5. Evaluation

**Figure 5:**
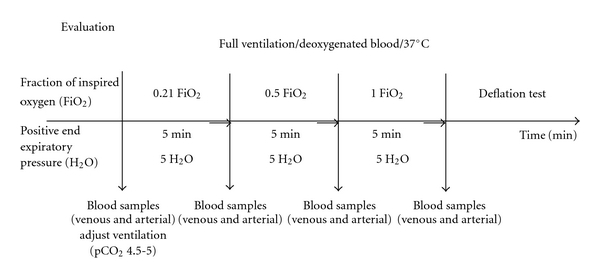
Photograph of the preparation of the donor lungs for *ex vivo* treatment showing cannulation of the pulmonary artery.

Figure 5 shows a flowchart describing lung evaluation, during which the lung is fully ventilated and the perfusate blood is deoxygenated with a mixture containing 93% nitrogen and 7% CO_2_ (i.e., the oxygenator is used to deoxygenate the perfusate), mimicking normal venous blood gas in the perfusate to the pulmonary artery, to be able to test the lung function. The PEEP was maintained at 5 cm H_2_O, except when it was temporarily increased to 8 cm H_2_O to eliminate atelectasis.

The gases entering the oxygenator were mixed to obtain perfusate gas values of pCO_2_ = 4.5–5 kPa. At this point, the rate of perfusion flow was maintained at 5-6 L/min (i.e., full flow). Samples of blood were taken before and after passing through the lungs (i.e., venous and arterial blood samples) to measure blood gases after 5 minutes' exposure to FiO_2_ of 0.21, 0.5, and, 1.0. 

A deflation test was then performed, which consists of observing the lung after the endotracheal tube is suddenly disconnected from the ventilator. In a normal lung, the whole lung should be deflatied (global atelectasis). If the deflation test was normal and the PO_2_ at an FiO_2_ of 1.0 was 50 kPa or higher, the lungs were accepted for transplantation.

The duration of reconditioning and evaluation in the *ex vivo* circuit was between 1 and 2 hours.

## 6. Cooling and Preservation

When reconditioning and evaluation of the lungs had been completed, and the surgeon had deemed them acceptable for transplantation according to the criteria described above, the lungs were cooled and preserved until the time of transplantation [13, 14]. The temperature of the ingoing perfusate was reduced to 25°C, and when the temperature had stabilized, perfusion was stopped. The pulmonary artery cannula and the trachea were clamped with the lungs in a semi-inflated state (FiO_2_ = 1.0). The lungs were then immersed in the perfusate, to which a buffered Perfadex solution was added. The extracorporeal circuit was then used to perfuse the solution through the box containing the immersed lungs, keeping the medium oxygenated and cooled at 8°C.

## 7. Transplantation and Outcome

After *ex vivo* reconditioning, the initially rejected donor lungs were transplanted into the patients. Double sequential lung transplantation was performed, with clam shell incision in four patients, and bilateral thoracotomy in the other two. Three patients were females and three were males. Three of the patients had been diagnosed with COPD, one patient with cystic fibrosis (CF), one with lung fibrosis, and one with *α*1-antitrypsin deficiency. The recipients had a median age of 54 years (range 35–64 y) at the time of transplantation [10]. The median height was 170 cm (range 158–180 cm), and the median weight 76 kg (range 55–94 kg) [10].

The donors had a median age of 59 years (range 34–63 y), a median height of 173 cm (range 154–182 cm), and a median weight of 77 kg (range 42–90 kg). The cause of death was cerebral bleeding in four of the donors, and two donors were resuscitated to spontaneous circulation after cardiac arrest [10]. A total of nine double lungs were reconditioned *ex vivo*. Six of them had PaO_2_ > 50 kPa at a FiO_2_ of 1.0 after reconditioning *ex vivo*. These six double lungs were transplanted, and the other 3 were not used. All donor lungs showed a normal chest radiograph prior to harvesting. The median PaO_2_ of the donors of the six successfully reconditioned lungs was 21.1 kPa (range 11.5–28.7 kPa) at FiO_2_ = 1.0 before harvesting. The corresponding values after *ex vivo* reconditioning were 68.7 kPa (range from 51.6 to 79.5) [10]. The median reconditioning time was 1 hour 29 minutes (range, 1 hour 6 minutes to 2 hours 1 minute) [10]. The time from harvesting to connection to the *ex vivo* reconditioning circuit was 6.4 hours (range 5.2–9.6 h), during which the lungs were stored in cold Perfadex solution. The total median time from lung harvesting to transplantation was 16.4 hours (range 10.6–17.5 h) for the right lung and 18.0 hours (range 12.6–21.5 h) for the left lung [10]. 

Three of the six patients were reintubated after extubation, and one of these had to be reintubated a second time. The six patients were connected to a respirator for a median time of 191 hours (range 25–2370 h). The patients remained in the intensive care unit for a median of 13 days (range 4–99 d), and the median length of hospital stay was 52 days (range 45–99 d) [11].

Three-month survival was 100%. One patient died due to sepsis, after 95 days, and one due to the rejection of the transplant after 9 months. The four patients surviving to the 12-month control showed increased 6-minute walking test values compared with the 3-month control, while the forced expiratory volume of air during 1 second increased in 3 and was the same in 1 patient. These four recipients are alive and well, without any signs of bronchiolitis obliterans syndrome, 24 months after the transplantation [10].

## 8. Comments

In the present paper, we present our clinical experience from the first six patients undergoing double lung transplantation, with *ex vivo* reconditioned donor lungs initially considered unacceptable for transplantation. We also present the reconditioning method and evaluation in detail with flowchart. All the lungs had been rejected by transplantation centers, both national and international, due to low PaO_2_ (<40 kPa) after 5 minutes' ventilation with 5 PEEP using 100% oxygen. The lungs were reconditioned and evaluated immediately upon arrival at the department. If arrival time was before 11 pm, reconditioning and evaluation were performed followed by immediate double lung transplantation. Lungs arriving after 11 pm were reconditioned and evaluated, and were then cooled, and transplantation was started at 8 am on the following day to avoid night-time surgery. 

We primed the system with STEEN solution and blood to a hematocrit of 15%, since all preclinical studies performed on animals by Professor Stig Steen at our department have been carried out with this kind of priming. When performing the first clinical trials on humans, we believed it advisable to use the same priming as in the preclinical studies [7, 8]. It may be possible to prime the machine in other ways with other solutions, or blood, but we have no such data. 

In patients where the total period of lung ischemia (before reconditioning, reconditioning, evaluation, and after evaluation) exceeded 17-18 hours, we found that even if the criteria for extubation regarding normal chest roentgenogram and excellent blood gases were fulfilled one to two days after surgery, they were not ready for extubation. If extubation was performed at this time, re-intubation and connection to a ventilator were often necessary a few hours later. We believe that half the fluid balance must be withdrawn from patients in this situation, and that one must wait at least two days after surgery, in addition to applying the conventional criteria for extubation, before extubation and disconnection from the ventilator are possible. We found that postoperative chest roentgenogram of the reconditioned lungs showed slightly increased vessel width and a tendency towards edema, which may indicate some kind of membrane injury in the lung parenchyma although the blood gas values were comparable to those in lungs not subjected to reconditioning prior to transplantation. 

Double lung transplantation was carried out in all six patients, in order to be able to evaluate the reconditioning procedure. In single lung transplantation, blood may become oxygenated by the remaining native lung, making it difficult to evaluate the success of transplanting reconditioned lungs.

All lung donors were free from pulmonary disease, and were not using any medication. The main reason for initial rejection of the lungs by the transplantation team was poor blood gas levels. Poor oxygenation in brain-dead donors connected to a ventilator is often the result of pulmonary edema, atelectasis, lung emboli or aspiration. All these conditions are well-known causes of a rapid decrease in oxygenation capacity.

With the *ex vivo *reconditioning and evaluation technique described here, we were able to “treat” pulmonary edema and eliminate atelectasis. It was also possible to carry out careful suction in the bronchus to minimize the effects of aspiration. 

Using the technique described in the present paper, we were able to “treat” pulmonary edema and atelectasis in the donor lungs and reevaluate their suitability for transplantation. The technique had not previously been used clinically on humans, and we increased the limit for acceptance for transplantation from the golden standard of PO_2_ > 40 kPa to >50 kPa after 5 minutes of ventilation with 5 PEEP. There was no evidence for this increased level, but it was adopted to increase the safety margin.

Based on our findings and experience, we would like to make the following two important recommendations to other cardiothoracic transplantation centers considering such an *ex vivo* lung reconditioning process to increase the number of donor lungs available for transplantation.

- Perform the transplantation immediately, or as soon as possible, after reconditioning and evaluation to limit the duration of ischemia. We encourage a careful attitude towards increasing ischemic time to an already marginal donor lung. At the same time, it would be an advantage, to both the surgeon and the patient, if night-time surgery could be avoided. Promising results have been obtained from experimental studies in pigs, where it was shown that the lungs and their vascular function could be safely preserved for up to 24 h [13], that the gas exchange system of the lungs can tolerate 1 h of warm ischemia after circulatory arrest without significant loss of functional capacity, and that the pulmonary artery can withstand warm ischemia for 3 h after death without impairment of endothelium-dependent relaxation or vascular smooth-muscle function [15]. Furthermore, it has been demonstrated that simple topical cooling of nonventilated lungs provides excellent preservation conditions for 12–24 h [13, 14]. However, long-term followup of a larger patient cohort is necessary before this method can become a clinical routine.
- Do not extubate and disconnect the patient from the ventilator during the first 24 hours after surgery, even if the chest X-ray appears normal, and the blood gases are excellent, that is, the criteria for extubation are fulfilled. We strongly recommend waiting 2-3 days, and ensuring that at least half of the fluid balance is withdrawn before extubation is performed.

In normal clinical lung transplantation, poor posttransplantation function remains a major problem, with significantly impaired function in 10% to 20% of cases [16]. It is, therefore, of the utmost importance to identify these lungs in advance, especially when using marginal donors. This method may be useful in the evaluation of marginal donor lungs as well as lungs from non-heart-beating donors. Furthermore, this method allows the time between harvest and transplantation to be increased, thereby allowing better planning for transplantation. Among other things, this will allow for the optimization of donor-recipient matching, for example, with HLA (human leukocyte antigen). We believe that this *ex vivo* reconditioning and evaluation model provides a valuable addition to the armamentarium for finding acceptable lungs in a donor population with inferior PaO_2_ values. However, as always, donor selection comes down to sound clinical judgment based on all the available data.
